# Pulmonary hypertension: the hallmark of acute COVID-19 microvascular angiopathy?

**DOI:** 10.1183/23120541.00389-2022

**Published:** 2023-02-06

**Authors:** Sri Harsha Dintakurti, Sanjana Kamath, Ciara Mahon, Suveer Singh, Bhavin Rawal, Simon P.G. Padley, Anand Devaraj, Laura C. Price, Sujal R. Desai, Tom Semple, Carole A. Ridge

**Affiliations:** 1 Imperial College London, London, UK; 2 Department of Imaging, Royal Brompton Hospital, London, UK; 3 Department of Adult Intensive Care, Royal Brompton Hospital, London, UK; 4 Pulmonary Hypertension Service, Royal Brompton Hospital, London, UK; 5 National Heart and Lung Institute, Imperial College London, London, UK; 6 The Margaret Turner-Warwick Centre for Fibrosing Lung Disease, Imperial College London, London, UK

## Abstract

There have been >481 million cases of COVID-19, caused by SARS-CoV-2, worldwide since December 2019 [1]. One of the hallmark features of acute COVID-19 pneumonia is pulmonary vascular involvement, most commonly manifesting as pulmonary artery thrombosis (PAT) [2, 3]. *Post mortem* data from 10 patients with COVID-19 pneumonia showed their central pulmonary arteries were free of thrombosis but all patients had small, firm thrombi in the peripheral parenchyma [4]. These findings raise the possibility that the computed tomography (CT) finding of isolated subsegmental PAT may reflect “the tip of the iceberg”; that small segmental thrombi may reflect downstream *in situ* thrombosis in the microvasculature. In patients with severe COVID-19 pneumonitis, dual-energy computed tomography pulmonary angiography (CTPA) has been used to demonstrate reduced pulmonary perfusion in the absence of any visible central thromboembolism [5, 6], further supporting the view that microscopic PAT is prevalent [6].

## Abstract 2

***In situ* pulmonary arterial thrombosis in COVID-19 is not visible on CTPA. However, the presence of CT-measured right heart and pulmonary artery dilatation in COVID-19 is likely attributable to this process and may be a possible surrogate for its detection.** https://bit.ly/3g7z5TV

*To the Editor:*

There have been >481 million cases of COVID-19, caused by SARS-CoV-2, worldwide since December 2019 [1]. One of the hallmark features of acute COVID-19 pneumonia is pulmonary vascular involvement, most commonly manifesting as pulmonary artery thrombosis (PAT) [2, 3]. *Post mortem* data from 10 patients with COVID-19 pneumonia showed their central pulmonary arteries were free of thrombosis but all patients had small, firm thrombi in the peripheral parenchyma [4]. These findings raise the possibility that the computed tomography (CT) finding of isolated subsegmental PAT may reflect “the tip of the iceberg”; that small segmental thrombi may reflect downstream *in situ* thrombosis in the microvasculature. In patients with severe COVID-19 pneumonitis, dual-energy computed tomography pulmonary angiography (CTPA) has been used to demonstrate reduced pulmonary perfusion in the absence of any visible central thromboembolism [5, 6], further supporting the view that microscopic PAT is prevalent [6].

The microscopic presence of extensive *in situ* thrombosis is thought to account for the development of pulmonary hypertension in patients with acute COVID-19 pneumonia [7]. This has been confirmed invasively in some cases, and more often using echocardiography, with evidence for raised pulmonary vascular resistance and, not infrequently, right ventricular radial dysfunction [8]. Determining the extent of thrombosis and its relationship with the development of pulmonary hypertension in patients with COVID-19 is a challenge, but it may enable earlier identification and intervention.

We hypothesise that PAT – both microscopic and macroscopic – is more common in acute COVID-19 pneumonia than in influenza pneumonia. We therefore sought 1) to establish whether isolated peripheral PAT is associated with pulmonary hypertension, and 2) to compare the incidence of CT features of pulmonary hypertension in both COVID-19 and influenza infection.

This retrospective, observational, single-centre study was approved by the National Health Service Health Research Authority (approval number 20/HRA/1434).

Single- and dual-energy CTPA of age- and gender-matched patients with influenza and COVID-19 pneumonia, referred for extra-corporeal membrane oxygenation (ECMO) and/or mechanical ventilation to the adult intensive care unit (AICU), from January 2016 to January 2021, were retrospectively evaluated. From both disease cohorts, those who had an available CTPA scan on the day of their admission to AICU were considered. Eligible influenza patients were then age- and gender-matched with the COVID-19 patients. After exclusion, 25 COVID-19 and 25 influenza patients were assessed (seven females and 18 males each). No patient had a pre-existing history of pulmonary hypertension prior to COVID-19/influenza infection.

The incidence of PAT, including central and peripheral, was recorded using CTPA. Furthermore, the incidence of peripheral pulmonary artery thrombi occurring in the absence of either central pulmonary embolism or deep vein thrombosis (DVT) was recorded (*i.e.* isolated peripheral pulmonary artery thrombi), as an imaging surrogate for *in situ* thrombosis. This qualitative CTPA analysis was assessed by two observers with 15 and 10 years’ experience of CT analysis, respectively. They reviewed CT scans in a blinded manner and further quantified PAT burden using the Qanadli scoring system [9]. DVT incidence was obtained through previous Doppler ultrasonography lower limb reports.

CT signs of pulmonary hypertension, such as cardiac dimensions, were assessed using Aquarius iNtuition Viewer (Terarecon, version 4.4.13.P3A). The incidence of PAT, severity of PAT extent, and CT cardiac dimensions, pulmonary and aortic diameter, and central pulmonary artery volume were measured and compared between the two groups. Blood D-dimer levels taken on admission were collected and assessed for correlation with CT signs of pulmonary hypertension and PAT burden in each cohort (GraphPad Prism 9).

Our final age- and gender-matched study cohort comprised 50 patients (COVID-19 pneumonia, n=25; influenza pneumonia, n=25). Both cohorts consisted of 28% females (n=7) and 72% males (n=18). Mean±sd age was 49.5±11.1 and 49.4±11.1 years in the COVID-19 and influenza cohorts, respectively. There were no statistically significant differences in age, gender or body mass index between groups (table 1).

**TABLE 1:** Demographic information, clinical characteristics, pulmonary artery thrombus incidence and computed tomography (CT) parameters of pulmonary hypertension of the COVID-19 and influenza study cohorts (n=50)

	**COVID-19**	**Influenza**	**p*-*value**
**Demographics and clinical characteristics**			
Age (years)	49.5±11.1	49.4±11.1	0.99
BMI (kg·m^−2^)	27.6±4.8	30.0±6.5	0.14
Female	7 (28%)	7 (28%)	
Male	18 (72%)	18 (72%)	
Pre-existing diabetes	4 (16%)	3 (12%)	0.68
Time on ECMO (days)	22.7±17.5	18.2±18.3	0.28
Time on mechanical ventilation (days)	27.4±15.3	9.5±3.5	0.08
APACHE II score	15.7±5.8	16.8±6.7	0.57
Murray score	2.9±0.4	2.7±0.6	0.27
**PA thrombi**			
Incidence of PA thrombi	15 (60%)	11 (44%)	0.40
Incidence of isolated peripheral PA thrombi	11 (44%)	8 (32%)	0.56
**CT parameters of pulmonary hypertension**			
RV/LV diameter ratio	1.16±0.37	0.90±0.13	0.008*
RA/LA diameter ratio	1.76±1.51	1.42±0.30	0.45
PA/Ao diameter ratio	1.04±0.16	0.71±0.37	0.0003^#^
PA volume (cm^3^)	67.3±14.8	59.4±16.6	0.08
Infarct volume (%)	0.28±0.93	0.19±0.53	0.88

Data are presented as mean±sd unless otherwise stated. Demographic information and clinical characteristics data were collected retrospectively, using IntelliSpace Critical Care and Anaesthesia software. To collect overall and isolated peripheral pulmonary artery (PA) thrombi data: segmental and subsegmental filling defects retrospectively observed on computed tomography pulmonary angiography (CTPA) were used as an imaging surrogate for peripheral PA thrombi. Data on central PA thrombi and deep vein thrombosis incidence were then collected retrospectively from previous CTPA and lower-limb Doppler ultrasound reports, respectively. CT parameters of pulmonary hypertension were measured retrospectively from CT images of the heart, which were reconstructed into a four-chamber view using Aquarius iNtuition Viewer. Data were analysed using either Student's t-test, Mann–Whitney U-test or Fisher's exact test as appropriate. BMI: body mass index; ECMO: extracorporeal membrane oxygenation; APACHE: Acute Physiology and Chronic Health Evaluation; RV: right ventricle; LV: left ventricle; RA: right atrium; LA: left atrium; Ao: aorta. *: p<0.05; ^#^: p<0.0005.

There was no significant difference in thrombotic burden between the COVID-19 and influenza cohorts. While the incidence of PAT was higher in COVID-19 compared to influenza infection, this difference did not reach statistical significance (60% *versus* 44%, respectively; p=0.3961). Interestingly, the incidence of isolated peripheral PAT (*i.e.* in absence of central pulmonary embolism or DVT, as a surrogate marker for *in situ* PAT) was similar between COVID-19 and influenza infection (44% *versus* 32%, p=0.5607) (table 1).

Despite this, CT signs of pulmonary hypertension were greater in COVID-19: right-to-left ventricular diameter ratio (1.16 *versus* 0.898, p=0.00786) and pulmonary artery to aorta diameter ratio (1.04 *versus* 0.705, p=0.000265) were higher in COVID-19 compared to influenza pneumonia (table 1). This trend persisted when the data were subgrouped by gender but did not reach statistical significance for both parameters, likely due to small sample size.

Levels of blood D-dimer showed no correlation to CT signs of pulmonary hypertension in either cohort; however, they did show a significant, moderate positive correlation with PAT burden in the COVID-19 cohort only (p=0.0035).

CT signs of pulmonary hypertension are more prevalent in COVID-19 compared to influenza infection but this is not attributable to PAT observable on CT. Given *post mortem* evidence of small pulmonary capillary occlusion by microthrombi [10], this suggests that pulmonary arterial microthrombi too small to visualise on CT might be responsible. Furthermore, it is possible that pulmonary vessel endothelialitis, also observed *post mortem*, may contribute to pulmonary hypertension in COVID-19 through release of vasoconstrictive inflammatory mediators [10].

Blood D-dimer levels were shown to be correlated to visible PAT burden in COVID-19 only. They may be useful in assessing the extent of PAT in severe COVID-19 and determining the requirement for anticoagulation in these patients early.

Our study does reiterate the high absolute prevalence of PAT seen in severe COVID-19 [11, 12]; however, existing literature also reports a higher PAT incidence (both overall and peripherally) in COVID-19 compared to influenza pneumonia patients receiving ECMO [13]. In contrast, we report not only similar incidences between COVID-19 and influenza, but also much higher rates of isolated peripheral PAT overall, particularly in the influenza cohort (we report 32% compared to 5% reported by Doyle *et al.* [13]). These differences may be explained by our age and gender cohort-matching process, which was not employed in the compared study, as the age profile of patients with severe influenza tends to be lower than in COVID-19 infection. Additionally, in contrast to other studies [14], all our influenza patients had severe acute respiratory failure (SARF) and were intubated, akin to the respiratory status of our COVID-19 patients.

A notable strength of this study is that, to the best of our knowledge, it is the first to compare isolated peripheral PAT in severe COVID-19 SARF patients requiring ECMO or mechanical ventilation to an age- and gender-matched cohort of severe non-COVID-19 pneumonia SARF patients.

In summary, we demonstrate that the likely process of *in situ* PAT in COVID-19 is not visible on CTPA. However, the presence of CT-measured right heart and pulmonary artery dilatation in COVID-19 is likely attributable to this process and so they may be a possible surrogate for its detection. Going forward, functional imaging such as perfusion scintigraphy and dual-energy CT should be investigated, as they may allow earlier detection in these patients prior to pulmonary hypertension CT markers developing.
